# Association of Advanced Glycation End Products With Lower-Extremity Atherosclerotic Disease in Type 2 Diabetes Mellitus

**DOI:** 10.3389/fcvm.2021.696156

**Published:** 2021-09-10

**Authors:** Lingwen Ying, Yun Shen, Yang Zhang, Yikun Wang, Yong Liu, Jun Yin, Yufei Wang, Jingrong Yin, Wei Zhu, Yuqian Bao, Jian Zhou

**Affiliations:** ^1^Department of Endocrinology and Metabolism, Shanghai Jiao Tong University Affiliated Sixth People's Hospital, Shanghai Clinical Center for Diabetes, Shanghai Key Clinical Center for Metabolic Disease, Shanghai Diabetes Institute, Shanghai Key Laboratory of Diabetes Mellitus, Shanghai, China; ^2^Anhui Institute of Optics and Fine Mechanics, Hefei Institutes of Physical Science, Chinese Academy of Sciences, Hefei, China; ^3^Science Island Branch, Graduate School of USTC, Hefei, China

**Keywords:** advanced glycation end products, lower extremity atherosclerotic disease, non-invasive, type 2 diabetes, biomaker

## Abstract

**Aims:** Advanced glycation end products (AGEs) were reported to be correlated with the development of diabetes, as well as diabetic vascular complications. Therefore, this study aimed at investigating the association between AGEs and lower-extremity atherosclerotic disease (LEAD).

**Methods:** A total of 1,013 type 2 diabetes patients were enrolled. LEAD was measured through color Doppler ultrasonography. The non-invasive skin autofluorescence method was performed for AGEs measurement. Considering that age plays an important role in both AGEs and LEAD, age-combined AGEs, i.e., AGE_age_ index (define as AGEs × age/100) was used for related analysis.

**Results:** The overall prevalence of LEAD was 48.9% (495/1,013). Patients with LEAD showed a significantly higher AGE_age_ (*p* < 0.001), and the prevalence of LEAD increased with ascending AGE_age_ levels (*p* for trend < 0.001). Logistic regression analysis revealed that AGE_age_ was significantly positively associated with risk of LEAD, and the odds ratios of presence of LEAD across quartiles of AGE_age_ were 1.00, 1.72 [95% confidence interval (CI) = 1.14–2.61], 2.72 (95% CI = 1.76–4.22), 4.29 (95% CI = 2.69–6.85) for multivariable-adjusted model (both *p* for trend < 0.001), respectively. The results were similar among patients of different sexes, body mass index, and with or without diabetes family history. Further, AGE_age_ presented a better predictive value for LEAD than glycated hemoglobin A_1c_ (HbA_1c_), with its sensitivity, specificity, and area under the curve of 75.5% (95% CI = 71.6–79.2%), 59.3% (95% CI = 54.9–63.6%), and 0.731 (0.703–0.758), respectively.

**Conclusion:** AGE_age_, the non-invasive measured skin AGEs combined with age, seems to be a more promising approach than HbA_1c_ in identifying patient at high risk of LEAD.

## Introduction

Lower-extremity atherosclerotic disease (LEAD), defined as a buildup of fatty deposits in peripheral vascular (i.e., atherosclerosis) that leads to progressive narrowing of the lower-extremity arteries, is reported to be the primary manifestation of peripheral arterial disease (PAD) (1). Several researches have reported the close relationships between LEAD and cardiac–cerebral vascular events (both non-fatal and fatal) including gangrene, amputation, and death (2, 3). Diabetes is an established important risk factor for LEAD since the prognosis of LEAD is worse in patients with diabetes than those without (4, 5).

LEAD may be silent or present with a variety of symptoms and signs indicative of extremity ischemia, such as claudication and rest pain (6, 7). However, given the common concurrence of neuropathy in patients with diabetes, LEAD often remains clinically imperceptible until the symptoms become aggravated and advance to ulceration or gangrene due to the loss of pain sensation (8). Therefore, early identification and intervention of LEAD should be highlighted to delay its progression and effectively reduce the risk of the related adverse outcomes, thereby improving patients' quality of life.

Doppler ultrasound examination of atherosclerotic stenosis or occlusive lesions of the lower extremities is an importantly auxiliary method for LEAD diagnosis. However, considering the time-consuming and the risk of omissions of plaque due to widely distributed lower-extremity arteries, there is an urgent need for an indicator for early detection of early-stage LEAD, especially in patients with diabetes.

Advanced glycation end products (AGEs) are modifications of proteins or lipids that become non-enzymatically glycated and oxidized (9, 10). AGEs affect nearly every type of cell and molecule in the body and are thought to be one factor in aging, as well as a causative role in diabetic vascular complications (11). Besides, considering the influence of age on LEAD, AGE_age_ index, defined as AGEs × age/100, was constructed to investigate the association of AGE_age_ with LEAD in patients with type 2 diabetes mellitus. In addition, whether AGE_age_ can be used for early screening of patients at high risk of LEAD was also analyzed.

## Subjects, Materials, and Methods

### Study Population

Individuals diagnosed with type 2 diabetes who were admitted to the Department of Endocrinology and Metabolism, Shanghai Jiao Tong University Affiliated to Sixth People's Hospital during May 2017 to November 2019 were recruited. Type 2 diabetes mellitus were diagnosed based on 1999 World Health Organization (WHO) criteria (12). Inclusion criteria include (1) age ≥ 18 years with the presence of type 2 diabetes mellitus; (2) a stable glucose-lowering regimen for the previous 3 months; (3) with valid data on both AGEs assessed by skin autofluorescence and lower limb ultrasound results; and (4) without any megascopic presence of dermopathy. Patients with diabetic ketoacidosis or severe and recurrent hypoglycemic events within the previous 3 months, and prior history of cardiovascular diseases, stroke, malignancy, mental disorders, or severe kidney, or liver dysfunction were excluded. Finally, 1,013 participants were enrolled into the final analysis.

This study was approved by the Shanghai Jiao Tong University Affiliated Sixth People's Hospital Ethics Committees and was in accordance with the Helsinki Declaration principles. Written informed consent was obtained from each participant.

### Assessment of Covariates

Family history of diabetes, medical history, smoking status (current smoker or not), and current medication therapy including glucose-lowering drugs, antihypertensive drugs, lipid-lowering drugs, and aspirin were recorded by self-report at baseline interview. Physical examination including height, weight, and blood pressure were performed in each patient. Body mass index (BMI) was calculated as weight (kg) divided by the square of height (m). Fasting venous blood sample was obtained after a 10-h overnight fasting. Biochemical measurements including glycated hemoglobin A_1c_ (HbA_1c_), glycated albumin (GA), fasting plasma glucose (FPG), fasting C-peptide (FCP), C-reactive protein (CRP), total cholesterol (TC), triglycerides (TG), high-density lipoprotein cholesterol (HDL-c), and low-density lipoprotein cholesterol (LDL-c) were assayed as previously reported (13).

### Assessment of AGEs

The spectroscopy device (Hefei Institutes of Physical Science, Chinese Academy of Sciences), mainly consisting of an ultraviolet light source, a broadband light source, a trifurcated fiber optic probe, and a compact charge coupled device spectrometer, was used to assess the skin AGEs. This device uses an excitation light with peak wavelength at 370 nm, which excites the AGEs in the skin that have fluorescence properties in a wavelength range of 420–600 nm. Besides, skin diffuse reflectance in a wavelength range of 350–600 nm was also detected to correct tissue absorption and scattering. The measurements were performed by trained nurses (at room temperature in a semidark environment) for three times at a normal skin site of the left volar side of the arm, and the average value was calculated for the analysis. AGE_age_ was defined as AGEs × age/100.

### Assessment of LEAD

Color Doppler ultrasonography was used for lower limb artery examination using an Acuson Sequoia 512 scanner (Siemens Medical Solutions, Mountain View, CA, USA) equipped with a linear array transducer with frequencies of 5–13 MHz. Seven arteries including femoral artery, deep femoral artery, superficial femoral artery, popliteal artery, anterior tibial artery, posterior tibial artery, and peroneal artery in each lower limb were measured for atherosclerotic plaque. The definition of atherosclerotic plaque have been described in detail previously, i.e., a focal structure encroaching into the arterial lumen ≥ 0.5 mm, 50% of the surrounding intima-media thickness (IMT) value, or an IMT thickness ≥ 1.5 mm (14–16). The presence of atherosclerotic plaques in any of the lower limb artery segments listed above was defined as LEAD (17).

### Statistical Analysis

All statistical analyses were performed using the SPSS Statistics, version 24.0 (SPSS, Inc., Chicago, IL), MedCalc 19.0.4 (MedCalc Software bvba, Ostend, Belgium) and SAS for windows version 9.3 (SAS Institute, Inc., Cary, NC). An unpaired Student's *t*-test was used for continuous variables with normal distributions for comparisons between groups. For continuous variables with skewed distributions, Wilcoxon rank sum test and Mann–Whitney *U*-test was conducted. The χ^2^ test was used for intergroup comparisons of categorical data. The linear regression model was used to examine the independent influencing factors of AGE_age_. Binary logistic regression analysis was used to assess the association between quartiles of AGE_age_ and LEAD. The receiver operating characteristic curve was used to evaluate the efficacy of AGE_age_ and HbA_1c_ in early detection of LEAD. A restricted cubic spline nested in logistic models was performed to test whether there was a dose–response or non-linear association of AGE_age_ as a continuous variable with the odds of LEAD. A two-tailed *p*-value of < 0.05 was considered statistically significant.

## Results

The clinical characteristics of the 1,013 enrolled type 2 diabetes patients (598 male, 415 female) were listed in Table 1. The mean age was 60 (53–66) years, and the mean diabetes duration was 11 (6-17) years. LEAD was detected in 495 participants, resulting in an overall prevalence of 48.9%. Patients with LEAD were older, more likely to be male, along with longer diabetes duration, higher systolic blood pressure (SBP) and AGEs, and lower diastolic blood pressure (DBP) (all *p* < 0.05). Moreover, the percentage of hypertension history and current smoker, as well as the percentage of patients receiving antihypertension, lipid-lowering, and anti-coagulant medications increased significantly (all *p* < 0.05).

**Table 1:** Clinical characteristics of study participants by the presence of LEAD.

	**Total** ***N* = 1,013**	**Without LEAD** ***N* = 518**	**With LEAD** ***N* = 495**	***P***
Male/Female	598/415	280/238	318/177	0.001
Age, years	60 (53–66)	56 (46–62)	63 (58–69)	<0.001
Duration, years	11 (6–17)	10 (4–15)	14 (9–20)	<0.001
BMI, kg/m^2^	24.5 (22.6–27.1)	24.7 (22.7–27.6)	24.4 (22.6–26.7)	0.097
SBP, mmHg	130 (120–140)	130 (120–140)	130 (120–142)	0.021
DBP, mmHg	80 (70–85)	80 (74–88)	80 (70–85)	0.003
HbA_1c_, %	8.5 (7.2–10.1)	8.5 (7.1–10.2)	8.4 (7.3–9.9)	0.472
GA, %	21.3 (17.6–26.5)	21.3 (17.5–26.2)	21.4 (17.6–26.8)	0.623
FPG, mmol/L	7.4 (6.1–9.1)	7.4 (6.2–9.1)	7.4 (5.9–9.0)	0.286
FCP, ng/ml	1.70 (1.11–2.44)	1.68 (1.12–2.41)	1.72 (1.11–2.47)	0.572
TC, mmol/L	4.60 (3.87–5.37)	4.69 (3.96–5.40)	4.55 (3.79–5.35)	0.120
TG, mmol/L	1.44 (1.03–2.16)	1.50 (1.03–2.29)	1.38 (1.03–2.03)	0.061
HDL-c, mmol/L	1.05 (0.88–1.27)	1.05 (0.87–1.26)	1.05 (0.88–1.27)	0.683
LDL-c, mmol/L	2.74 (2.13–3.40)	2.76 (2.15–3.38)	2.70 (2.12–3.43)	0.614
CRP, mg/L	0.83 (0.38–1.75)	0.84 (0.38–1.87)	0.83 (0.38–1.66)	0.640
AGEs	76.1 (70.2–82.6)	73.8 (68.9–80.0)	78.6 (72.4–84.9)	<0.001
AGE_age_	45.8 (37.7–53.0)	41.2 (32.5–48.8)	49.3 (43.2–56.5)	<0.001
Diabetes family history, *n* (%)	606 (59.8)	307 (59.3)	299 (60.4)	0.712
Hypertension history, *n* (%)	513 (50.6)	216 (41.7)	297 (60.0)	<0.001
Current smoker, *n* (%)	245 (24.2)	111 (21.4)	134 (27.1)	0.036
**Anti-diabetic agents**, ***n*****(%)**				
Oral anti-diabetes drugs	697 (68.8)	357 (68.9)	340 (68.7)	0.936
Metformin	455 (44.9)	243 (46.9)	212 (42.8)	0.206
Sulfonylureas	224 (22.1)	108 (20.8)	116 (23.4)	0.326
Thiazolidinediones	70 (6.9)	36 (6.9)	34 (6.9)	0.993
Glinides	68 (6.7)	33 (6.4)	35 (7.1)	0.707
DPP-4 inhibitors	110 (10.9)	57 (11.0)	53 (10.7)	0.920
Glycosidase inhibitors	375 (37.0)	175 (33.8)	196 (39.6)	0.104
SGLT-2 inhibitors	5 (0.5)	2 (0.4)	3 (0.6)	0.680
GLP-1 receptor agonists	15 (1.5)	9 (1.7)	6 (1.2)	0.606
Insulin	705 (69.6)	348 (67.2)	357 (72.1)	0.088
**Antihypertension agents**, ***n*****(%)**	468 (46.2)	189 (36.5)	279 (56.4)	<0.001
ACE inhibitors	12 (1.2)	6 (1.2)	6 (1.2)	1.000
ARBs	336 (33.2)	129 (24.9)	207 (41.8)	<0.001
CCBs	235 (23.2)	104 (20.1)	131 (26.5)	0.017
β-Blockers	106 (10.5)	46 (8.9)	60 (12.1)	0.101
Diuretics	78 (7.7)	31 (6.0)	47 (9.5)	0.045
**Lipid-lowering agents**, ***n*****(%)**	657 (64.9)	296 (57.1)	361 (72.9)	<0.001
Statins	589 (58.1)	249 (48.1)	340 (68.7)	<0.001
Fibrates	74 (7.3)	51 (9.8)	23 (4.6)	0.002
Ezetimibe	2 (0.2)	1 (0.2)	1 (0.2)	1.000
Aspirin, *n* (%)	305 (30.1)	107 (20.7)	198 (40.0)	<0.001

*Data are expressed as median with interquartile range or n (%)*.

*ACE, angiotension-converting enzyme; ARB, angiotensin receptor blockers; CCB, calcium channel blockers; DPP-4, dipeptidyl peptidase 4; GLP-1, glucagon like peptide-1; SGLT-2, sodium–glucose cotransporter-2*.

**Figure 1:**
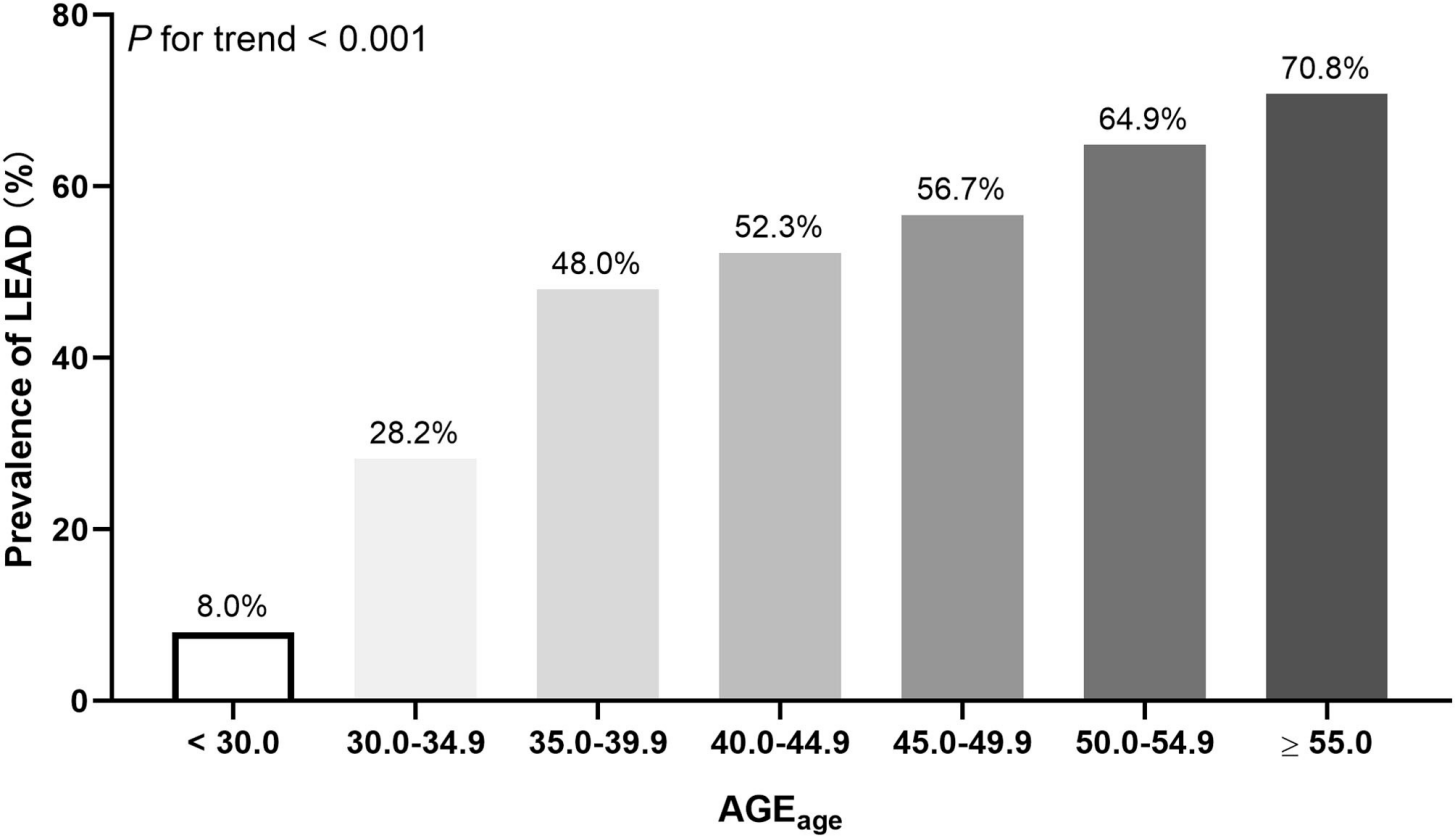
The prevalence of LEAD stratified by AGE_age_ levels.

Besides, we also found an increase in AGE_age_ in patients with LEAD compared with those without LEAD [49.3 (43.2–56.5) vs. 41.2 (32.5–48.8), *p* < 0.001]. Similarly, as shown in Figure 1, the prevalence of LEAD increased progressively across the categories of increasing AGE_age_ (*p* for trend < 0.001). Then, the participants were stratified according to AGE_age_ levels (AGE_age_ < 43.2 and AGE_age_ ≥ 43.2). Supplementary Table 1 depicts the characteristics of subjects by AGE_age_ levels.

Multivariate linear regression analysis defined the AGE_age_ levels as the dependent variable, and sex, diabetes duration, BMI, SBP, family history of diabetes, HbA_1c_, FPG, lipid profile, CRP, smoking status, antidiabetic therapy, antihypertensive medication, lipid-lowering medication, and aspirin use were designated as the independent variables. The results showed an independently positive association between diabetes duration, SBP, HDL-c, antihypertensive medication, lipid-lowering medication, and AGE_age_ (all *p* < 0.01). Besides, BMI and TC were negatively associated with the AGE_age_ levels (all *p* < 0.001).

Logistic regression analysis revealed that AGE_age_ was significantly positively associated with risk of LEAD, that is, the odds ratios of presence of LEAD across quartiles of AGE_age_ were 1.00, 2.43 [95% confidence interval (CI) = 1.66–3.57], 4.71 (95% CI = 3.21–6.92), and 7.95 (95% CI = 5.34–11.84) for crude model, and 1.00, 1.72 (95% CI = 1.14–2.61), 2.72 (95% CI = 1.76–4.22), and 4.29 (95% CI = 2.69–6.85) for multivariable-adjusted duration, BMI, SBP, DBP, hypertension history, current smoker, use of angiotensin receptor blockers, calcium channel blockers, diuretics, statins, fibrates, and aspirin model (both *p* for trend <0.001), respectively. The results were similar among patients of different sexes, BMI, and with or without diabetes family history (Table 2).

**Table 2:** Association of AGE_age_ with LEAD among patients with diabetes.

	**AGE**_**age**_				***P* for trend**	**Per SD increase**
	**<37.7**	**37.7–45.8**	**45.9–52.8**	**≥52.9**		
**Total**						
No. of patients	253	254	253	253		
No. of cases	59	108	149	179		
Odds ratios	1.00	2.43 (1.66–3.57)	4.71 (3.21–6.92)	7.95 (5.34–11.84)	<0.001	2.50 (2.13–2.92)
Multivariable-adjusted odds ratios	1.00	1.72 (1.14–2.61)	2.72 (1.76–4.22)	4.29 (2.69–6.85)	<0.001	2.00 (1.66–2.42)
**Male**						
No. of patients	184	128	138	148		
No. of cases	48	62	93	115		
Odds ratios	1.00	2.66 (1.65–4.29)	5.86 (3.61–9.51)	9.87 (5.94–16.41)	<0.001	2.80 (2.28–3.43)
Multivariable-adjusted odds ratios	1.00	1.63 (0.97–2.76)	2.69 (1.53–4.71)	4.64 (2.54–8.48)	<0.001	2.10 (1.64–2.69)
**Female**						
No. of patients	69	126	115	105		
No. of cases	11	46	56	64		
Odds ratios	1.00	3.03 (1.45–6.35)	5.01 (2.39–10.50)	8.23 (3.87–17.50)	<0.001	2.35 (1.79–3.07)
Multivariable-adjusted odds ratios	1.00	2.39 (1.08–5.29)	3.28 (1.45–7.43)	4.82 (2.05–11.34)	0.001	1.92 (1.40–2.61)
**BMI < 25.0**						
No. of patients	121	145	145	154		
No. of cases	25	57	80	114		
Odds ratios	1.00	2.49 (1.43–4.32)	4.73 (2.73–8.18)	10.94 (6.20–19.33)	<0.001	2.84 (2.25–3.58)
Multivariable-adjusted odds ratios	1.00	1.89 (1.05–3.41)	3.00 (1.63–5.51)	6.64 (3.43–12.84)	<0.001	2.41 (1.84–3.16)
**BMI ≥ 25.0**						
No. of patients	132	109	108	99		
No. of cases	34	51	69	65		
Odds ratios	1.00	2.53 (1.47–4.36)	5.10 (2.93–8.87)	5.51 (3.12–9.74)	<0.001	2.25 (1.81–2.80)
Multivariable-adjusted odds ratios	1.00	1.90 (1.02–3.52)	3.08 (1.59–5.98)	3.05 (1.53–6.07)	<0.001	1.78 (1.36–2.32)
**Without diabetes family history**						
No. of patients	117	100	87	103		
No. of cases	26	44	54	72		
Odds ratios	1.00	2.75 (1.53–4.95)	5.73 (3.10–10.59)	8.13 (4.44–14.90)	<0.001	2.42 (1.91–3.06)
Multivariable-adjusted odds ratios	1.00	2.61 (1.37–4.98)	4.64 (2.26–9.52)	5.73 (2.76–11.90)	<0.001	2.12 (1.59–2.82)
**With diabetes family history**						
No. of patients	136	154	166	150		
No. of cases	33	64	95	107		
Odds ratios	1.00	2.22 (1.34–3.68)	4.18 (2.54–6.87)	7.77 (4.58–13.17)	<0.001	2.56 (2.06–3.18)
Multivariable-adjusted odds ratios	1.00	1.25 (0.71–2.19)	1.91 (1.08–3.39)	3.26 (1.75–6.09)	<0.001	1.89 (1.46–2.44)
**Age < 65.0**						
No. of patients	253	245	187	58		
No. of cases	59	100	102	32		
Odds ratios	1.00	2.27 (1.54–3.34)	3.95 (2.62–5.94)	4.05 (2.24–7.33)	<0.001	1.07 (1.05–1.09)
Multivariable-adjusted odds ratios	1.00	1.66 (1.08–2.54)	2.24 (1.40–3.61)	2.28 (1.17–4.44)	<0.001	1.05 (1.03–1.07)
**Age ≥ 65.0**						
No. of patients	-	8	67	195		
No. of cases	-	7	48	147		
Odds ratios	-	1.00	0.36 (0.04–3.13)	0.44 (0.05–3.65)	0.718	1.02 (0.99–1.06)
Multivariable-adjusted odds ratios	-	1.00	0.30 (0.03–2.86)	0.32 (0.03–2.95)	0.577	1.02 (0.98–1.06)

*SD, standard deviation*.

*Adjusted for duration, BMI, SBP, DBP, hypertension history, current smoker, use of ARB, CCB, diuretics, statins, fibrates, and aspirin other than the variable for stratification*.

**Figure 2:**
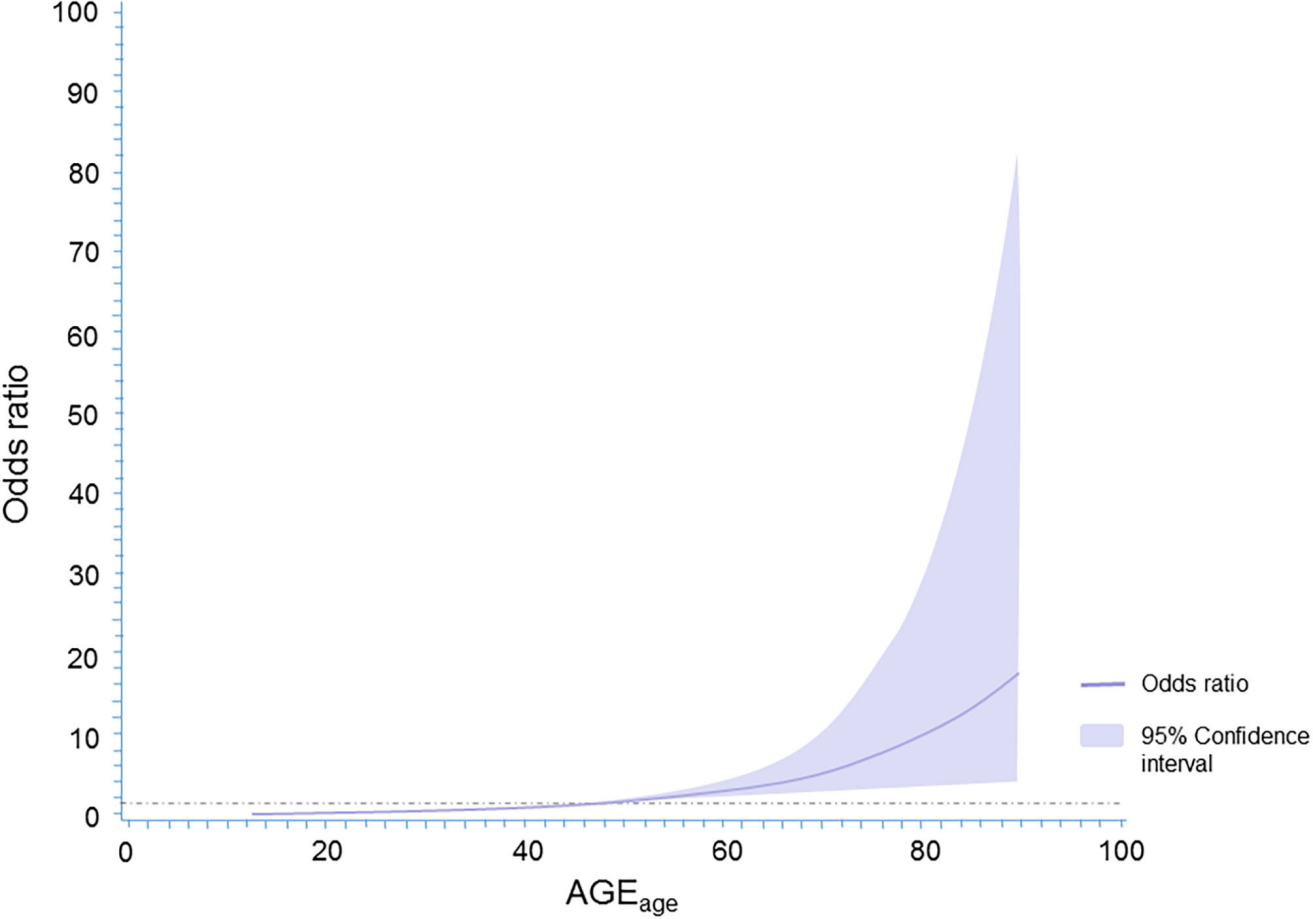
The spline association between AGE_age_ and LEAD in patients with type 2 diabetes mellitus. Adjustments were made for sex, diabetes duration, BMI, blood pressure, lipid panels, HbA_1c_, CRP, family history of diabetes, smoking status, antidiabetic therapy, antihypertensive medication, lipid-lowering medication, and aspirin use.

When AGE_age_ was considered as a continuous variable by using restricted cubic splines, a graded positive association of AGE_age_ with the odds of presence of LEAD was observed (*p* for trend < 0.001; Figure 2). This curve trend was consistent with the findings in Table 2 when AGE_age_ was considered as a categorical variable.

When stratified by sexes, BMI, HbA_1c_, never and past or current smokers, as well as taking antidiabetic, lipid-lowering, antihypertensive, and antiplatelet medication or not, the graded positive association between AGE_age_ and the odds of presence of LEAD were consistent across all subgroups. Subgroup analyses were then performed to examine potential effect modifiers, and we observed an interaction of HbA_1c_ and using antiplatelet medication or not with a *p* for interaction <0.01 (Supplementary Table 2). Supplementary Table 3 described the confounding factors associated with LEAD.

Areas under the curve (AUCs) of AGE_age_ and HbA_1c_ for early detection of LEAD were measured. The results showed that AGE_age_ had a better predictive value for LEAD than HbA_1c_, concretely, 0.731 (0.703–0.758) for AGE_age_ and 0.513 (0.482–0.544) for HbA_1c_, respectively (*p* < 0.01). The optimal cutoff point for AGE_age_ in early detecting LEAD was 43.2, with a sensitivity of 75.5% (95% CI = 71.6–79.2%) and a specificity of 59.3% (95% CI = 54.9–63.6%). The study participants were stratified based on sex, BMI, diabetes family history, and current smoker or not, and the results showed an acceptable efficacy of AGE_age_ < 43.2 in early identifying LEAD in all related subgroups. In addition, the efficacy of AGE_age_ in early detection of LEAD seems more pronounced in male and BMI < 25 kg/m^2^ subgroup type 2 diabetic patients (Table 3).

**Table 3:** The efficacy of AGE_age_ ≥ 43.2 in early identifying LEAD in type 2 diabetic patients.

	**AUC**	**Sensitivity**	**Specificity**	**PPV**	**NPV**
Total	0.731 (0.703–0.758)	75.5 (71.6–79.2)	59.3 (54.9–63.6)	65.0 (62.3–67.6)	70.8 (67.1–74.1)
**Sex**					
Male	0.768 (0.732–0.801)	73.8 (68.7–78.5)	68.5 (62.6–74.0)	73.6 (69.8–77.1)	68.8 (64.3–72.9)
Female	0.696 (0.650–0.740)	78.6 (71.9–84.3)	48.5 (41.9–55.1)	54.4 (50.7–58.0)	74.3 (68.0–79.8)
**BMI**					
<25	0.750 (0.712–0.785)	82.1 (77.1–86.4)	56.8 (50.9–62.7)	65.2 (61.8–68.4)	76.4 (71.2–80.9)
≥25	0.715 (0.670–0.756)	67.4 (60.9–73.5)	62.4 (55.7–68.8)	64.8 (60.3–69.1)	65.1 (60.1–69.8)
**Diabetes family history**					
Yes	0.721 (0.683–0.756)	77.0 (71.9–81.6)	55.2 (49.3–60.9)	63.5 (60.2–66.7)	70.3 (65.3–74.9)
No	0.748 (0.702–0.789)	73.3 (66.6–79.2)	65.4 (58.4–71.9)	67.6 (62.9–71.9)	71.3 (65.9–76.1)
**Current smoker**					
Yes	0.711 (0.650–0.767)	70.1 (61.7–77.6)	66.7 (56.9–75.4)	72.7 (66.7–78.1)	63.7 (56.8–70.1)
No	0.744 (0.711–0.774)	77.6 (73.0–81.7)	57.3 (52.3–62.2)	62.8 (59.8–65.7)	73.3 (69.1–77.2)

*NPV, negative predictive value; PPV, positive predictive value*.

## Discussion

In this observational study, the AGE_age_ index was proposed for the first time. We observed a positive association of AGE_age_ with the odds of presence of LEAD among patients with type 2 diabetes mellitus, independent of traditional risk factors for LEAD that include HbA_1c_. Besides, AGE_age_ may be a suitable indicator for early identification of patients at high risk of LEAD, with its optimal cutoff point of 43.2.

LEAD is an emerging public health burden with an endemic progression worldwide that affects over 200 million people worldwide (18). Related studies reported that LEAD was two to four times more frequent in people with type 2 diabetes than in the general population (19, 20), and the prevalence of LEAD also increased along with the rising diabetes duration as shown in the United Kingdom Prospective Diabetes Study (UKPDS), concretely, 1.2% when first diagnosed with diabetes, and increased up to 12.5% after 18 years of evolution (21). Besides, LEAD is particularly frequent in diabetic patients with worse outcomes, especially the risk of lower limb amputation, four to five times higher, compared with non-diabetic subjects (22, 23), suggesting that poor glycemic control may play an important role in LEAD progression.

HbA_1c_ is a well-established marker for assessment of glycemic control. In the UKPDS trial, each 1% reduction in HbA_1c_ was associated with a 43% reduction in the risk of major LEAD (amputation or death induced by peripheral vascular event) (24). However, HbA_1c_ is insufficient in terms of the overall evaluation of glycemic control, i.e., patients with similar HbA_1c_ could have totally distinct glucose profiles. The results of the ADVANCE trial demonstrated that the incidence of major LEAD (lower-limb ulceration, amputation, revascularization requirement, or death following a PAD) was comparable between intensive and standard glucose control groups (25, 26). Moreover, type 2 diabetic patients with or without LEAD in the current study share similar HbA_1c_ levels. Therefore, other factors related to glycemic control beyond HbA_1c_ may be related to LEAD.

AGEs are considered as one factor in aging and some age-related chronic diseases including Alzheimer's disease, cardiovascular disease, stroke, and diabetes. Studies have reported that hyperglycemic status may promote the accumulation of AGEs, while AGEs can cause vascular stiffening and entrapment of LDL particles in the artery walls by inducing crosslinking of collagen in the context of cardiovascular disease (27, 28). AGEs can also cause LDL glycation, thereby further promoting its oxidation, while oxidized LDL is one of the major factors in the development of atherosclerosis. In addition, the interaction between AGEs and RAGE (receptor for AGEs) induced oxidative stress, and activation of inflammatory pathways in vascular endothelial cells also plays an important role in the development of systemic atherosclerosis including LEAD (28). These findings raised the possibility that AGEs and AGE-generated indicators may be an alternative indicator reflecting LEAD.

Considering the fact that AGEs are closely correlated with age, while LEAD is reported to be discovered during the fifth decade of life and the prevalence of LEAD increased exponentially after 65 years (29), we proposed the AGE_age_ index for the first time, which combines AGEs and age organically. Consistent with our hypothesis, in the current study, the level of AGE_age_ was significantly increased in LEAD patients with type 2 diabetes mellitus. Accordingly, we also found a graded positive association of AGE_age_ with the odds of presence of LEAD, even after adjusting for clinical risk factors, including HbA_1c_, which imply the value of AGE_age_ in assessing the risk of diabetic complications independent of HbA_1c_. Particularly, skin autofluorescence is a non-invasive method for AGEs detection. All the above-mentioned suggest that AGE_age_ may be a simple and effective indicator for predicting LEAD.

Meanwhile, we proposed that in type 2 diabetes patients, AGE_age_ might be a more suitable indicator than HbA1c for mimicking the poor prognosis of diabetes, i.e., LEAD, regardless of gender, BMI, and family history of diabetes. Our results showed that AGE_age_ had a significantly higher predictive value for LEAD than HbA_1c_. With the cutoff point of 43.2, around 3/4 patients with LEAD (384/591) were successfully identified. Based on the current study, we recommend type 2 diabetic patients whose AGE_age_ ≥ 43.2 are considered at high risk for LEAD that needs ultrasound for further confirmation.

The relatively large sample size and well-documented clinical information of the current study makes our findings more reliable. Nevertheless, there are some limitations that should be pointed out. First, since the current study was a cross-sectional study, the cause-and-effect relationship between AGEs and LEAD could not be clarified. Second, the participants enrolled in the current study were Chinese type 2 diabetic hospitalized patients. Considering the racial difference in circulating AGEs, as well as the promotion of hyperglycemia on AGE accumulation, whether our results can be generalized to all diabetic patients and even patients with cardiovascular diseases needs further study.

## Conclusion

In conclusion, we provide evidence that AGE_age_ is associated with the prevalence of LEAD in patients with type 2 diabetes mellitus independent of HbA_1c_. AGE_age_, the non-invasive measurement of accumulated AGEs combined with age, seems a promising approach than HbA_1c_ to mimic the poor prognosis of hyperglycemia, i.e., triage for patient at high risk of LEAD.

## Data Availability Statement

The original contributions presented in the study are included in the article/Supplementary Material, further inquiries can be directed to the corresponding author/s.

## Ethics Statement

The studies involving human participants were reviewed and approved by the Shanghai Jiao Tong University Affiliated Sixth People's Hospital Ethics Committees. The patients/participants provided their written informed consent to participate in this study.

## Author Contributions

JZ and YiW designed the study. LY and YS collected the data. LY, YS, and YZ performed statistical analysis and wrote the paper. YuW, JiY, and WZ help with the AGEs measurement. JZ, YB, JuY, YL, and YiW revised the paper and contributed to discussion. LY, YS, and YZ had equal contribution to this paper and were the guarantors. All authors contributed to the article and approved the submitted version.

## Funding

This work was funded by the National Key R&D Program of China (2018YFC2000802), the Shanghai Municipal Education Commission—Gaofeng Clinical Medicine Grant Support (20161430), the Bureau of International Cooperation, Chinese Academy of Sciences (Grant no. 116134KYSB20170018), and the Natural Science Foundation of Anhui Province of China (1908085QH365).

## Conflict of Interest

The authors declare that the research was conducted in the absence of any commercial or financial relationships that could be construed as a potential conflict of interest. The reviewer NW has declared a shared affiliation, with no collaboration, with several of the authors LY, YS, JuY, YuW, JiY, WZ, YB, and JZ, to the handling editor at the time of review.

## Publisher's Note

All claims expressed in this article are solely those of the authors and do not necessarily represent those of their affiliated organizations, or those of the publisher, the editors and the reviewers. Any product that may be evaluated in this article, or claim that may be made by its manufacturer, is not guaranteed or endorsed by the publisher.
