# Supplementary material for: Association of Advanced Glycation End Products With Lower-Extremity Atherosclerotic Disease in Type 2 Diabetes Mellitus
Source: Front Cardiovasc Med. 2021 Sep 10;8:696156. doi: 10.3389/fcvm.2021.696156 (PMC8460767; doi:10.3389/fcvm.2021.696156)
Supplement: Supplementary file 1 [file Data_Sheet_1.docx]

Supplementary table 1. Clinical characteristics of study participants by AGE_age_ levels

|  | Total  N = 1013 | AGE_age_ < 43.2  N = 424 | AGE_age_ ≥ 43.2  N = 589 | *p* |
| --- | --- | --- | --- | --- |
| Male/Female | 598/415 | 272/152 | 326/263 | 0.005 |
| Age, yrs | 60 (53–66) | 51 (41–55) | 65 (61–69) | < 0.001 |
| Duration, yrs | 11 (6–17) | 9 (3–12) | 14 (10–20) | < 0.001 |
| BMI, kg/m^2^ | 24.5 (22.6–27.1) | 25.0 (23.0–27.8) | 24.2 (22.2–26.6) | < 0.001 |
| SBP, mmHg | 130 (120–140) | 130 (120–140) | 131 (120–144) | < 0.001 |
| DBP, mmHg | 80 (70–85) | 80 (75–90) | 80 (70–83) | < 0.001 |
| HbA_1c_, % | 8.5 (7.2–10.1) | 8.7 (7.3–10.3) | 8.4 (7.2–9.8) | 0.051 |
| GA, % | 21.3 (17.6–26.5) | 21.3 (17.5–27.0) | 21.3 (17.6–26.4) | 0.988 |
| FPG, mmol/L | 7.4 (6.1–9.1) | 7.6 (6.4–9.1) | 7.3 (5.8–9.0) | 0.008 |
| FCP, ng/mL | 1.70 (1.11–2.44) | 1.77 (1.11–2.43) | 1.66 (1.10–2.44) | 0.947 |
| TC, mmol/L | 4.60 (3.87–5.37) | 4.71 (4.03–5.47) | 4.53 (3.79–5.32) | 0.004 |
| TG, mmol/L | 1.44 (1.03–2.16) | 1.53 (1.06–2.47) | 1.37 (0.99–1.97) | < 0.001 |
| HDL-c, mmol/L | 1.05 (0.88–1.27) | 1.01 (0.83–1.21) | 1.08 (0.91–1.30) | < 0.001 |
| LDL-c, mmol/L | 2.74 (2.13–3.40) | 2.78 (2.25–3.47) | 2.69 (2.08–3.35) | 0.055 |
| CRP, mg/L | 0.83 (0.38–1.75) | 0.90 (0.41–1.94) | 0.74 (0.36–1.60) | 0.039 |
| Diabetes family history, *n* (%) | 606 (59.8) | 236 (55.7) | 370 (62.8) | 0.022 |
| Hypertension history, *n* (%) | 513 (50.6) | 165 (38.9) | 348 (59.1) | < 0.001 |
| Current smoker, *n* (%) | 245 (24.2) | 113 (26.7) | 132 (22.4) | 0.120 |
| LEAD, *n* (%) | 495 (48.9) | 123 (29.0) | 372 (63.2) | < 0.001 |
| Anti-diabetic agents, *n* (%) |  |  |  |  |
| Oral anti-diabetes drugs | 697 (68.8) | 288 (67.9) | 409 (69.4) | 0.608 |
| Insulin | 705 (69.6) | 291 (68.6) | 414 (70.3) | 0.572 |
| Anti-hypertension agents, *n* (%) | 468 (46.2) | 145 (34.2) | 323 (54.8) | < 0.001 |
| Lipid-lowering agents, *n* (%) | 657 (64.9) | 231 (54.5) | 426 (72.3) | < 0.001 |
| Aspirin, *n* (%) | 305 (30.1) | 90 (21.2) | 215 (36.5) | < 0.001 |

Data were expressed as median with interquartile range, or n (%).

Abbreviation: AGE_age_: advanced glycation end products × age / 100 index; BMI: body mass index; CRP: C-reactive protein; DBP: diastolic blood pressure; FCP: fasting C-peptide; FPG: fasting plasma glucose; GA: glycated albumin; HbA_1c_: glycated hemoglobin A_1c_; HDL-C: high density lipoprotein Cholesterol; LDL-C: low density lipoprotein Cholesterol; LEAD: lower extremity atherosclerotic disease; SBP: systolic blood pressure; TC: total cholesterol; TG: triglyceride;

Supplementary table 2. Subgroup analysis.

|  | AGE_age_ | | | | As a continuous variable |
| --- | --- | --- | --- | --- | --- |
|  | < 37.7 | 37.7–45.8 | 45.9–52.8 | ≥ 52.9 |  |
| Sex | | | | | |
| Male | 1.00 | 1.97 (1.14–3.41) | 3.86 (2.18–6.85) | 6.71 (3.61–12.49) | 2.06 (1.68–2.54) |
| Female | 1.00 | 2.44 (1.11–5.39) | 3.92 (1.75–8.79) | 5.10 (2.19–11.89) | 1.79 (1.39–2.32) |
| BMI | | | | | |
| < 25 | 1.00 | 2.14 (1.17–3.90) | 3.58 (1.94–6.59) | 7.40 (3.83–14.28) | 2.10 (1.69–2.62) |
| ≥ 25 | 1.00 | 2.75 (1.43–5.31) | 5.14 (2.61–10.16) | 4.39 (2.18–8.85) | 1.80 (1.44–2.25) |
| HbA_1c_ (%)^**^ | | | | | |
| < 8.5 | 1.00 | 1.86 (1.01–3.41) | 2.52 (1.35–4.71) | 4.43 (2.25–8.73) | 1.71 (1.37–2.13) |
| ≥ 8.5 | 1.00 | 2.32 (1.24–4.36) | 5.34 (2.76–10.32) | 6.66 (3.35–13.21) | 2.10 (1.67–2.65) |
| Diabetes family history | | | | | |
| No | 1.00 | 2.83 (1.44–5.60) | 6.42 (3.04–13.56) | 5.49 (2.63–11.49) | 1.81 (1.44–2.29) |
| Yes | 1.00 | 1.88 (1.06–3.35) | 2.91 (1.63–5.19) | 5.34 (2.83–10.07) | 1.97 (1.59–2.43) |
| Smoking status | | | | | |
| Never and past smoking | 1.00 | 2.26 (1.33–3.85) | 4.44 (2.59–7.60) | 6.58 (3.74–11.58) | 2.02 (1.69–2.43) |
| Current smoking | 1.00 | 2.80 (1.23–6.36) | 2.45 (1.00–5.97) | 3.69 (1.38–9.87) | 1.66 (1.20–2.28) |
| Using anti-diabetic medications | | | | | |
| Oral anti-diabetes drugs | 1.00 | 2.17 (1.29–3.65) | 3.91 (2.30–6.65) | 5.01 (2.84–8.86) | 1.86 (1.54–2.25) |
| Insulin | 1.00 | 2.36 (1.41–3.97) | 4.17 (2.44–7.12) | 7.12 (4.03–12.59) | 2.09 (1.73–2.52) |
| Using lipid-lowering medications | | | | | |
| No | 1.00 | 1.96 (0.91–4.24) | 6.21 (2.83–13.61) | 8.43 (3.48–20.39) | 2.38 (1.76–3.22) |
| Yes | 1.00 | 2.10 (1.23–3.59) | 2.71 (1.57–4.67) | 4.54 (2.54–8.11) | 1.72 (1.43–2.08) |
| Using anti-hypertensive medications | | | | | |
| No | 1.00 | 4.01 (2.22–7.25) | 5.43 (2.87–10.28) | 8.98 (4.42–18.24) | 2.29 (1.81–2.90) |
| Yes | 1.00 | 0.97 (0.48–1.93) | 2.36 (1.21–4.62) | 3.00 (1.52–5.93) | 1.59 (1.28–1.98) |
| Using Aspirin^**^ | | | | | |
| No | 1.00 | 2.90 (1.69–4.99) | 6.03 (3.40–10.69) | 9.24 (5.03–16.99) | 2.20 (1.81–2.69) |
| Yes | 1.00 | 0.86 (0.37–2.01) | 1.09 (0.47–2.54) | 1.71 (0.71–4.11) | 1.37 (1.03–1.81) |

Abbreviation: AGE_age_: advanced glycation end products × age / 100 index; BMI, body mass index; CRP: C-reactive protein; HbA_1c_, glycated hemoglobin A_1c_; SBP, systolic blood pressure.

*^*^, p* for interaction < 0.05; *^**^, p* for interaction < 0.01

Adjusted for sex, diabetes duration, BMI, SBP, lipid panels, HbA_1c_, CRP, family history of diabetes, smoking status, anti-diabetic therapy, anti-hypertensive medication, lipid-lowering medication and aspirin use other than the variable for stratification.

Supplementary table 3. Univariate analysis of confounding factors associated with LEAD.

|  | Odds ratios (95% confident intervals) | *p* |
| --- | --- | --- |
| Age | 1.09 (1.07–1.12) | < 0.001 |
| Duration, | 1.08 (1.06–1.10) | < 0.001 |
| BMI | 0.96 (0.92–0.99) | 0.015 |
| SBP | 1.01 (1.00–1.02) | 0.030 |
| DBP | 0.98 (0.97–0.99) | 0.001 |
| HbA_1c_ | 0.96 (0.91–1.02) | 0.225 |
| GA | 1.00 (0.98–1.02) | 0.983 |
| FPG | 0.99 (0.94–1.04) | 0.727 |
| FCP, | 1.04 (0.93–1.17) | 0.490 |
| TC | 0.93 (0.83–1.04) | 0.179 |
| TG | 0.95 (0.88–1.02) | 0.153 |
| HDL-c | 1.03 (0.68–1.55) | 0.902 |
| LDL-c | 0.98 (0.86–1.12) | 0.794 |
| CRP | 0.97 (0.90–1.04) | 0.377 |
| AGEs | 1.05 (1.04–1.07) | < 0.001 |
| AGE_age_ | 1.07 (1.06–1.09) | < 0.001 |
| Diabetes family history | 1.05 (0.82–1.35) | 0.712 |
| Hypertension history | 2.10 (1.63–2.70) | < 0.001 |
| Current smoker | 1.36 (1.02–1.82) | 0.036 |
| Anti-diabetic agents |  |  |
| Oral anti-diabetes drugs |  |  |
| Metformin | 0.85 (0.66–1.09) | 0.192 |
| Sulfonylureas | 1.16 (0.86–1.56) | 0.322 |
| Thiazolidinediones | 0.99 (0.61–1.61) | 0.959 |
| Glinides | 1.12 (0.68–1.83) | 0.656 |
| DPP-4 inhibitors | 0.97 (0.65–1.44) | 0.879 |
| Glycosidase inhibitors | 1.24 (0.96–1.60) | 0.097 |
| SGLT-2 inhibitors | 1.57 (0.26–9.46) | 0.620 |
| GLP-1 receptor agonists | 0.69 (0.25–1.96) | 0.491 |
| Insulin | 1.26 (0.97–1.65) | 0.088 |
| Anti-hypertension agents |  |  |
| ACE inhibitors | 1.05 (0.34–3.27) | 0.937 |
| ARBs | 2.17 (1.66–2.83) | < 0.001 |
| CCBs | 1.43 (1.07–1.92) | 0.016 |
| β-blockers | 1.42 (0.94–2.12) | 0.093 |
| Diuretics | 1.65 (1.03–2.64) | 0.038 |
| Lipid-lowering agents |  |  |
| Statins | 2.37 (1.83–3.06) | < 0.001 |
| Fibrates | 0.45 (0.27–0.74) | 0.002 |
| Ezetimibe | 1.05 (0.07–16.8) | 0.974 |
| Aspirin | 2.56 (1.94–3.38) | < 0.001 |

Abbreviation: AGEs: advanced glycation end products; AGE_age_: AGEs × age / 100 index; ACE: Angiotension converting enzyme; ARB: Angiotensin receptor blockers; BMI: body mass index; CCB: Calcium channel blockers; CRP: C-reactive protein; DBP: diastolic blood pressure; DPP-4: Dipeptidyl peptidase 4; FCP: fasting C-peptide; FPG: fasting plasma glucose; GA: glycated albumin; GLP-1: Glucagon like peptide-1; HbA_1c_: glycated hemoglobin A1c; HDL-c: high density lipoprotein Cholesterol; LDL-c: low density lipoprotein Cholesterol; LEAD: lower extremity atherosclerotic disease; SBP: systolic blood pressure; SGLT-2: Sodium-glucose co-transporter-2; TC: total cholesterol; TG: triglyceride.
